# Supplementary material for: Potential for Exposure to Particles and Gases throughout Vat Photopolymerization Additive Manufacturing Processes
Source: Buildings (Basel). Author manuscript; Available in PMC 2023 Nov 13. (PMC10641710; doi:10.3390/buildings12081222)
Supplement: Supplemental file [file NIHMS1943038-supplement-Supplemental_file.pdf]

Potential for Exposure to Particles and Gases throughout Vat Photopolymerization Additive Manufacturing Processes

Lauren N. Bowers, Aleksandr B. Stefaniak*, Alycia K. Knepp, Ryan F. LeBouf, Stephen B. Martin, Jr., Anand C. Ranpara, Dru A. Burns, and M. Abbas Virji

National Institute for Occupational Safety and Health, Morgantown, WV, 26505 USA

* Author to whom correspondence should be sent:

Aleksandr B. Stefaniak
National Institute for Occupational Safety and Health
1095 Willowdale Road
Morgantown, WV, 26505 USA
Tel.: +1-304-285-6302
E-mail: AStefaniak@cdc.gov

Supplemental File

Materials and Methods

Evacuated Cansiter Sample Analysis

Pressurized 450 mL canisters were concentrated prior to analysis using an autosampler (Model7016CD, Entech Instruments, Inc., Simi Valley, CA) with a 10 °C transfer line attached to a pre-concentrator (Model 7200, Entech Instruments, Inc.). The pre-concentrator was coupled with a 6890N/5973N gas chromatography – mass spectrometry (GC-MS) system (Agilent Technologies, Inc., Santa Clara, CA) with a RTX-1 capillary column 60 m long x 0.32 mm ID x 1 µm film thickness (Restek Corporation, Bellefonte, PA, USA). Pre-concentration conditions were: modified cold trap dehydration: module 1 (empty) at -20 °C, desorbed at 10 °C, and baked at 150 °C for 7 min; module 2 (glass beads) focused at -80 °C, desorbed at 180 °C, and baked at 190 °C; and module 3 (focuser) focused at -150 °C. GC conditions were: oven temperature program set to 35 °C for 2 min, followed by 8 °C/min ramp to 170 °C, then 20 °C/min ramp to a final temperature of 220 °C, which was held for 3 min; injector temperature was set to 250 °C with a 20:1 split (split flow 20.2 mL/min); detector temperature was 280 °C; and column flow rate was set to 1 mL/min. Mass spectrometer analysis conditions were: scan mode 35-350 amu; threshold at 150; scan speed at 2.84 scans/s; solvent delay to 4.5 min; source temperature at 230 °C; and quadrapole temperature at 150 °C. A one-point calibration check standard (10 ppb) and an instrument blank (ultra-high purity nitrogen gas) were analyzed with each set of samples within a 24-hour period. MSD Chemstation D.02.00.275 (Agilent Technologies., Inc.) was used for data acquisition. Final concentrations were calculated based on the response of the closest internal standard (bromochloromethane, 1,4-difluorobenzene, and chlorobenzene-d5). All quantitative measurement results were background corrected for the concentration of VOCs measured on background samples collected before the start of the tasks.

Analysis for Ethyl Methacrylate and Methyl Methacrylate

After sample collection, the front and back sorbent sections of XAD-2 tubes were put into separate 4 mL glass vials. The samples were chemically desorbed using 2 mL of carbon disulfide and were sonicated for 30 minutes. The samples were transferred to auto sampler vials and analyzed by GC/flame ionization detector (FID) (Thermo Analytical Trace 1310, Thermo Fisher Scientific, Waltham, MA, USA) with a Phenomenex ZB – 1 30 m x 0.32 mm ID and 1 µm film thickness column. Analysis conditions were: a flow rate at 2.8 mL/min at a constant flow; a split flow at 14 mL/min; and a continuous purge flow at 5

mL/min. The injection and detector temperature for the FID were 250 °C. Parameters for the oven were: initial temperature was 50 °C with a hold time of 3 minutes; oven rate was 10 °C/min with a hold temperature of 120 °C with a hold time of 0 minutes.

Analysis for 2-hydroxypropyl methacrylate, 2-hydroxyethyl methacrylate, and 2-hydroxyethyl acrylate

The front and back sorbent sections of collected charcoal tubes were placed into separate amber autosampler vials, which were chemically desorbed using 1.0 mL of 5% methanol in methylene chloride. Samples sat for 30 minutes with occasional agitation. The samples were prepared by removing a 100 µL aliquot which was transferred to a micro-vial insert. An internal standard consisting of naphthalene-d8 was added to each vial, briefly vortexed, and analyzed using a GC-MS in SCAN mode (Agilent Model 7890B GC-Agilent Model 5975C MS) with a Phenomenex ZB – 5MS, 30 m x 0.25 mm ID with a 0.25 µm film thickness column. The column had a constant flow rate of 1.5 mL/min. A splitless injection was used with an injection temperature at 280 °C and an injection volume of 1 µL. Oven parameters were: initial temperature was 50 °C with a hold time of 1.0 min. Temperature program rate was 20 °C/min with a hold temperature at 90 °C for 0.5 min. Final temperature program rate was 12 °C/min with a hold temperature at 280 °C for 0.4 min.

Mixed Cellulose Ester (MCE) Filter preparation

Filter samples were placed in a clean 50 mL polyethylene centrifuge tube. Each filter cassette was wiped out with an MCE filter and added to a sample tube. The sample tube was filled with 2.5 mL of 12.1 M hydrochloric aced and placed in a hot block and heated at a temperature of 95 °C for 15 min. Samples were removed, cooled, and diluted to a final volume of 25 mL with deionized water.

Results

Table S1. Elemental content of bulk feedstock grey resin

Metal	Concentration (mg/kg)[a]
Arsenic	(2.8)
Barium	<0.2
Cadmium	<0.3
Iron	<2
Molybdenum	<0.4
Phosphorous	160
Tin	59
Titanium	3.2
Zinc	(9.2)
Zirconium	0.72

[a] < = below analytical limit of detection (LOD); ( ) = value above LOD but below the analytical limit of quantification

Additional VOC results from canister samples (all in µg/m^3):

d-Limonene – DLP process: pouring task (9.5), printing task (1.7), recovery task (1.7), curing task (3.4)

Toluene – DLP process: curing task (2.4)

α-Pinene – DLP process: printing task (7.1)

m,p-Xylene – DLP process: curing task (2.2)

n-Hexane – SLA process: curing task (2.0)

Ethylbenzene – SLA and DLP processes: all concentrations below background

o-Xylene – SLA and DLP processes: all concentrations below background

Benzene – SLA and DLP processes: all values below the limit of detection (i.e., <0.64)

Methyl methacrylate – SLA and DLP processes: all values below the limit of detection (i.e., <1.33)

Additional VOC results from charcoal tube samples (all in μg/m^3):

2-Hydroxyethyl methacrylate – SLA and DLP processes: all values below the limit of detection (i.e., <28.4)

2-Hydroxyethyl acrylate – SLA and DLP processes: all values below the limit of detection (i.e., <33.7)

Additional VOC results from sorbent tubes containing XAD-2 resin samples (all in μg/m^3):

Ethyl methacrylate – SLA and DLP processes: all values below the limit of detection (i.e., <104.3)

Methyl methacrylate – SLA and DLP processes: all values below the limit of detection (i.e., <115.4)

Table S2. Background-corrected airborne concentrations of aerosol elemental constituents for select tasks. Values are geometric mean (µg/m^3) and (geometric standard deviation) unless noted otherwise.[a] Elements in *italics* were also detected in the bulk feedstock resin.

Task	Process[b]	Barium*	Chromium*	Iron*	Manganese*	*Tin*	*Titanium*	*Zinc**	*Zirconium*‡
Pour	SLA	<0.06	0.94 (1.3)[A]	1.26 – 1.52	0.13	0.89	0.04	0.59	0.03
	DLP	0.04 (1.3)[A]	0.91 (1.2)[A]	<2.37	<2.24	0.18 (3.5)[†]	0.07 (1.1)	0.36 (3.2)[A,ǂ]	0.07 (1.1)
Print	SLA	<0.02	0.09 (2.1)[B]	0.22 (2.0)[A,†]	0.001	<0.19	<0.0094	0.10 – 0.25	<0.01
	DLP	0.002	<0.04	<0.66	<0.62	<0.07	<0.0072	0.10 (1.6)[B,†]	<0.01
Recover	SLA	<0.06	0.85 (1.6)[A]	0.60 – 0.69	0.09 (1.8)[A]	<0.59	0.02	0.33 (1.4)[A,B†]	0.02
	DLP	0.04 (1.2)[A,ǂ]	0.43 – 0.91	<2.22	<2.10	0.12 – 0.17	0.07 (1.2)	0.11 – 0.19	0.07 (1.2)
Cure	SLA	<0.02	0.10 (2.2)[B]	0.20 (2.8)[A,†]	0.05 (2.3)[A]	0.19	0.001	0.04 (4.9)[A,B,†]	0.01 – 0.01
	DLP	<0.03	<0.10	0.75 – 2.72	0.90	0.17	<0.012	0.17 (2.2) [A,B,†]	0.08
Sand	SLA	0.04 – 0.05	0.30 (1.7)[B]	0.40 (1.8)[A,†]	0.01	<0.35	0.02	0.21 – 0.21	0.02 (1.4)[†]
	DLP	0.01 – 0.01	0.04 – 0.09	<1.32	<1.25	<0.12	0.01 (1.5)	0.22 (1.6)[A,B,†]	0.02 (1.3)

[a] < = all samples below limit of detection, single value = one sample result above background, range = two or three sample results above background

[b] SLA = stereolithography, DLP = digital light processing

* Adjusted regression models (differences among tasks are the same for both printers and there is no difference between printers for each task). Within each column, capital letters for GM values that are not connected by the same letter are significantly different ($p < 0.05$). For example, using Chromium data, the "Pouring[A]" and "Recovery[A]" tasks are significantly different than the "Printing[B]", "Curing[B]", and "Sanding[B]" tasks.

‡ Mixed regression models (significant interaction between printer and task). DLP process – pouring = recovery > sanding ($p < 0.05$).

⸸ One of five replicate values imputed using β-substitution method (see Method section)

† Two of five replicate values imputed using β-substitution method (see Method section)

Additional background-corrected elemental concentration results from filter samples (all in μg/m^3)

Arsenic – DLP process: recover task (0.65 – 0.95)

Cadmium – DLP process: pour task (0.10 – 0.11), recover task (0.05 – 0.8), sand task (0.002 – 0.03)

Lead – SLA process: print task (GM = 0.10, GSD = 1.4),[&] recover task (0.50)

Lead – DLP process: print task (0.09)

Lithium – DLP process: cure task (0.05)

Molybdenum – DLP process: pour task (0.10), print task (0.03 – 0.04), recover task (GM = 0.13, GSD = 1.2)[&]

Nickel – DLP process: sand task (0.14)

Strontium – DLP process: pour task (0.05), print task (0.01), cure task (0.01), sand task (0.02 – 0.03)

Tellurium – DLP process: recover task (1.51)

Vanadium – SLA process: cure task (0.11)

Vanadium – DLP process: pour task (0.46), recover task (0.43)

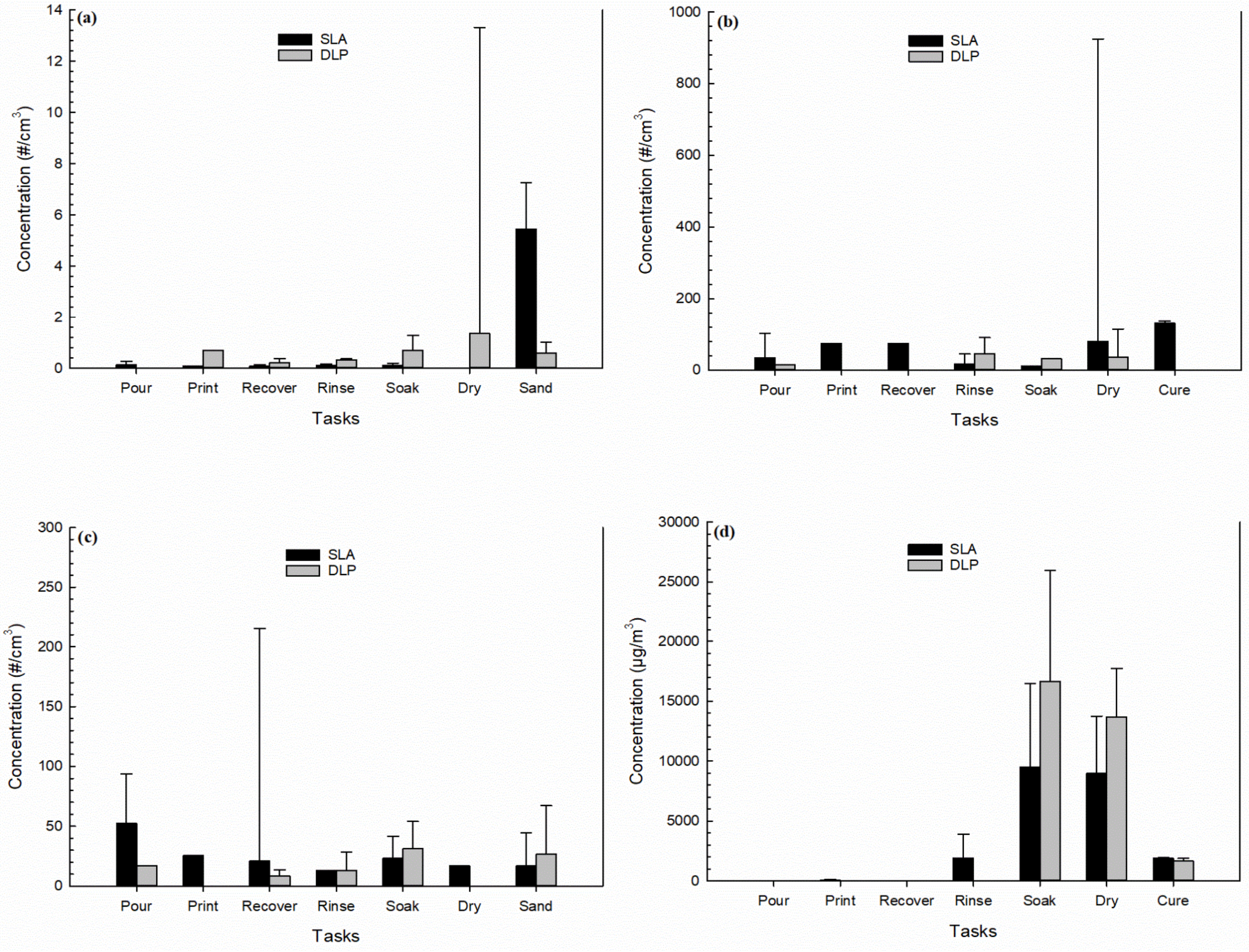

Figure S1. Untransformed average contaminant concentrations by process and task: (a) APS, (b) FMPS, (c) CNC, and (d) TVOC. Whiskers are 95% confidence intervals. Bars with no whiskers indicate one of five replicates had result above background.

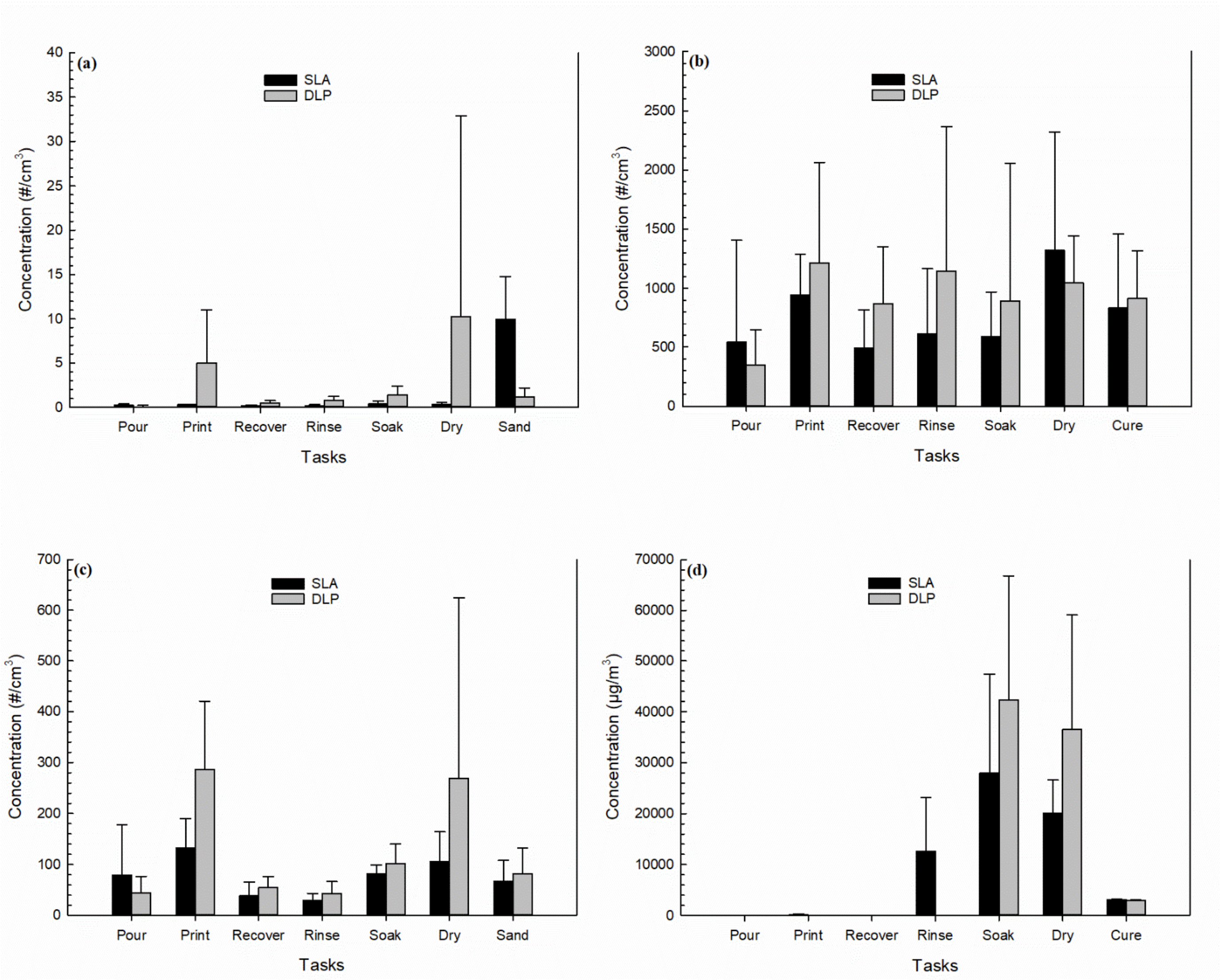

Figure S2. Untransformed maximum contaminant concentrations by process and task: (a) APS, (b) FMPS, (c) CNC, and (d) TVOC. Whiskers are 95% confidence intervals. Bars with no whiskers indicate one of five replicates had result above background

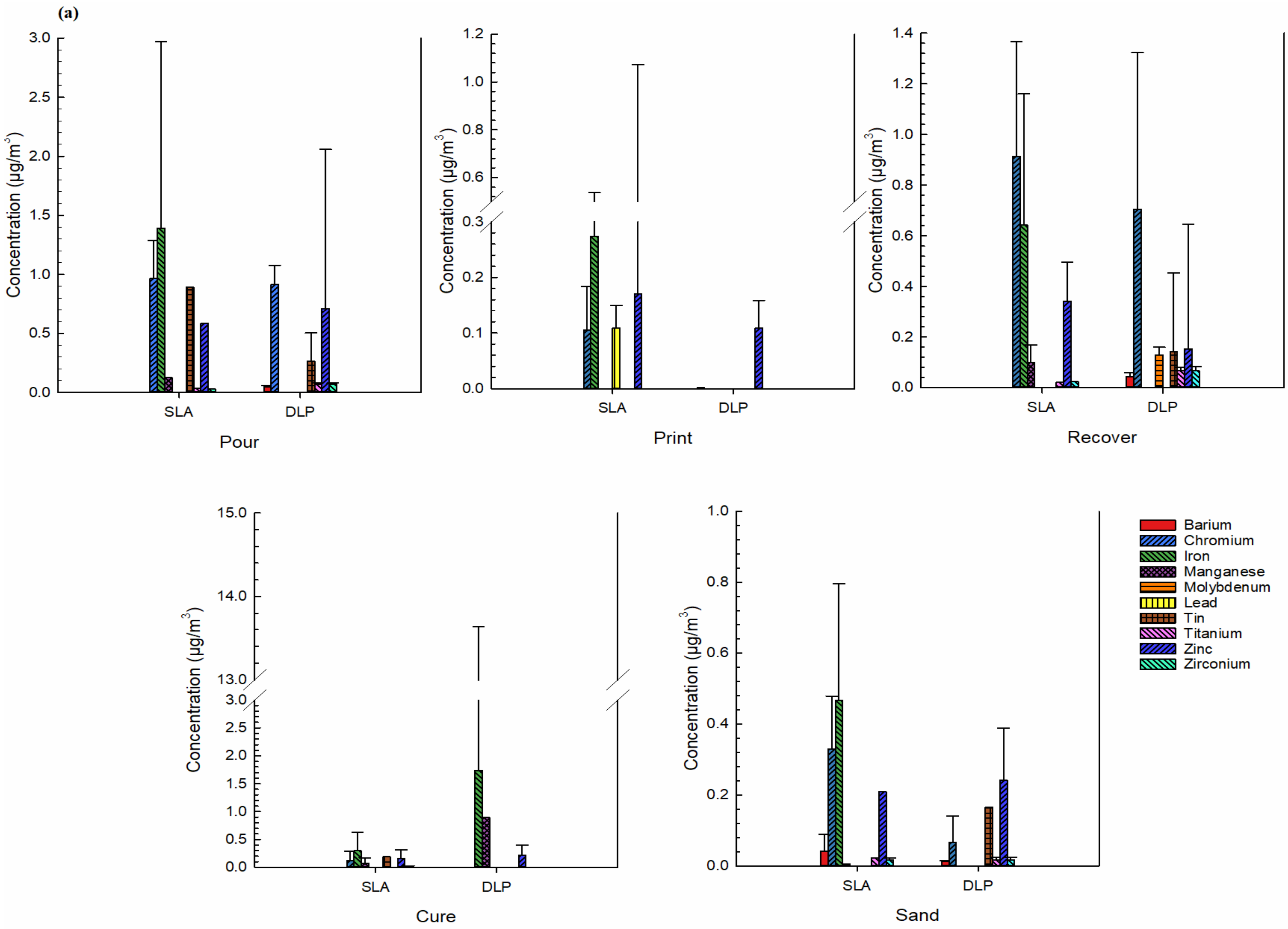

(a)
Concentration (µg/m3)
SLA
DLP
Pour
Print
Recover
Cure
Sand
Barium
Chromium
Iron
Manganese
Molybdenum
Lead
Tin
Titanium
Zinc
Zirconium

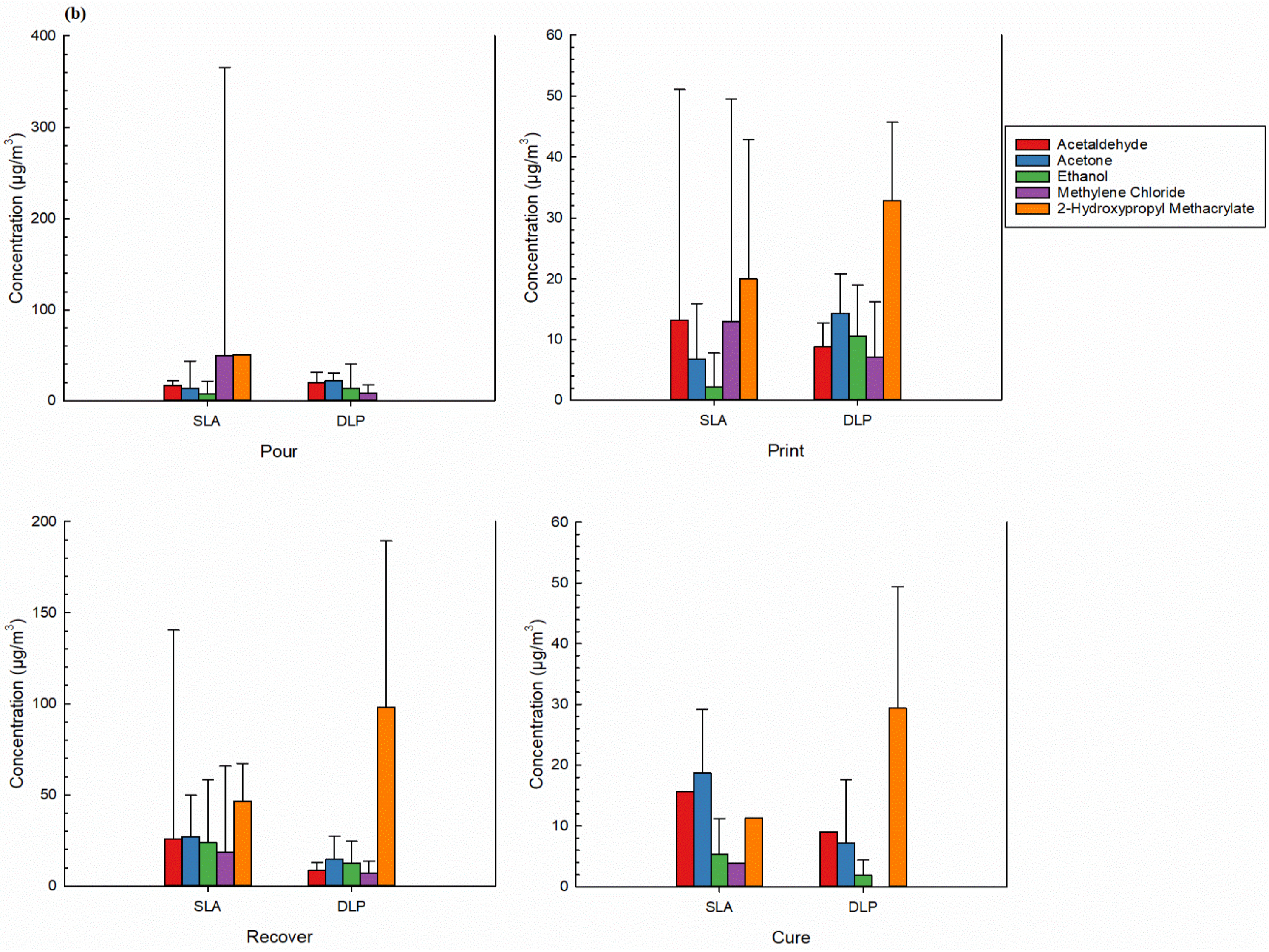

Figure S3. Untransformed average contaminant concentrations by process and task: (a) elements from MCE samples, and (b) specific VOCs from canister (acetaldehyde, acetone, ethanol, and methylene chloride) and charcoal (2-hydroxypropyl methacrylate) samples. Whiskers are 95% confidence intervals. Bars with no whiskers indicate one of five replicates had result above background

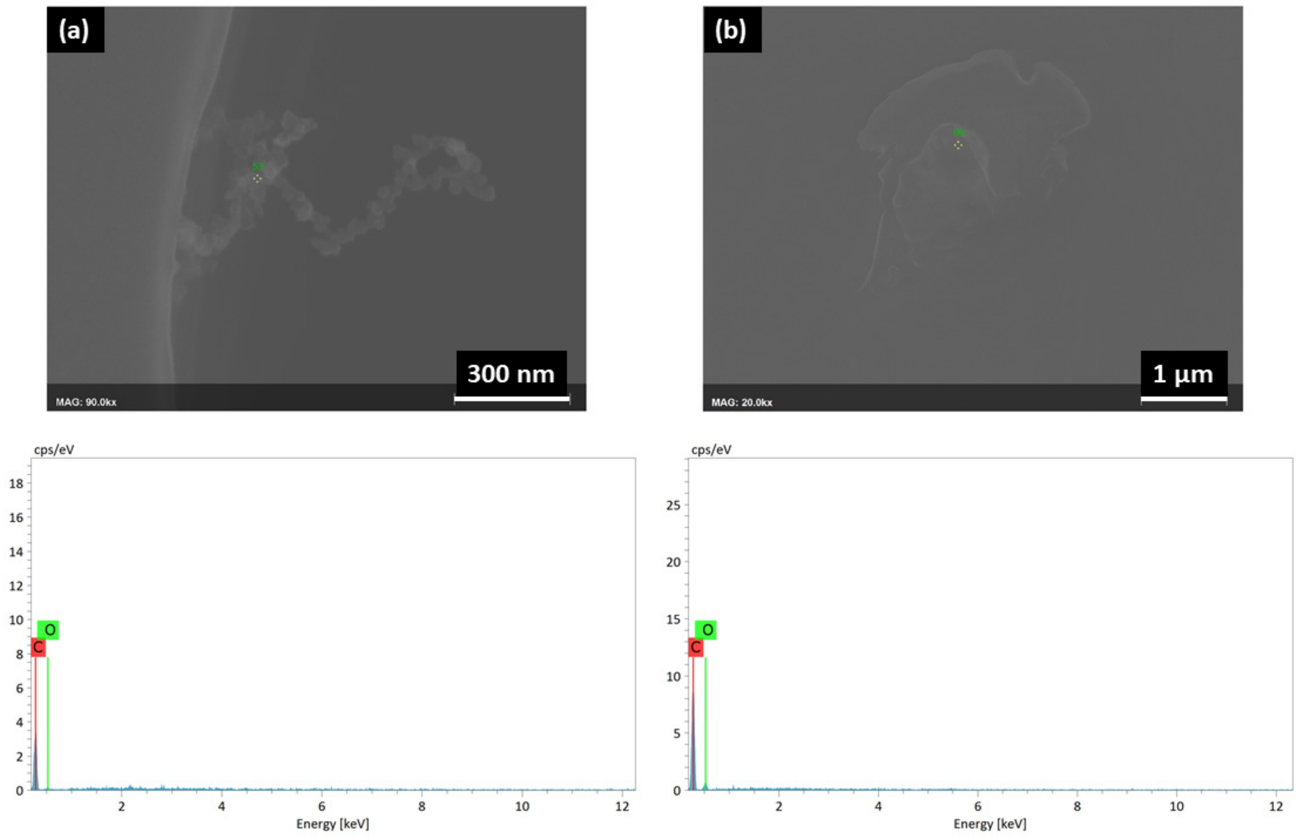

Figure S4. Scanning electron microscopy images and energy dispersive X-ray analysis spectra of particles released during the pouring task: (a) nanoscale, branch-chain particles (SLA process) and (b) micronscale, irregular-shaped particles (DLP process). These particles were composed of carbon.

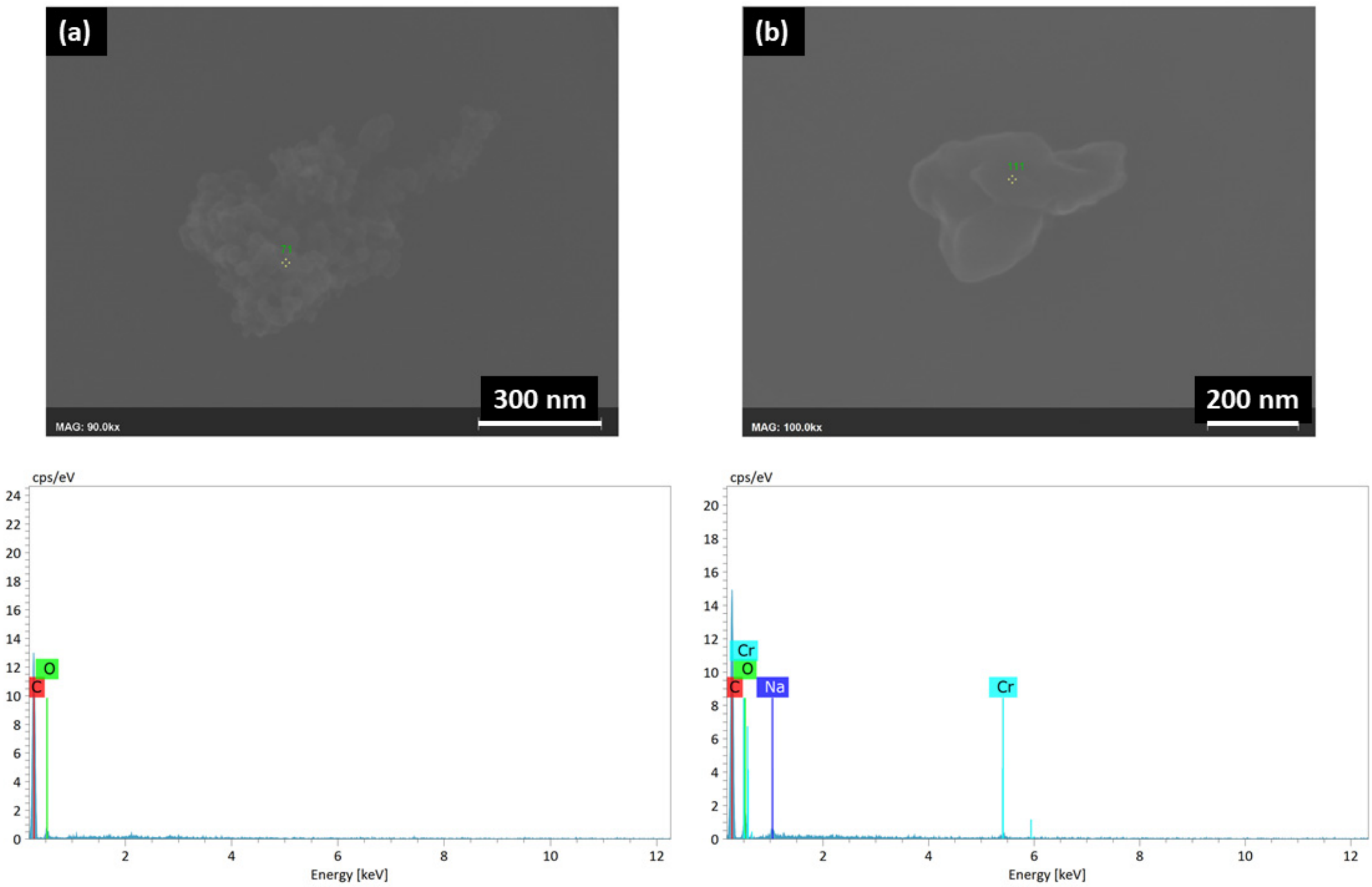

Figure S5. Scanning electron microscopy images and energy dispersive X-ray analysis spectra of particles released during the printing task: (a) nanoscale, branch-chain particles composed of carbon (SLA process) and (b) micronscale, irregular-shaped particle composed of carbon, sodium, and chromium (DLP process).

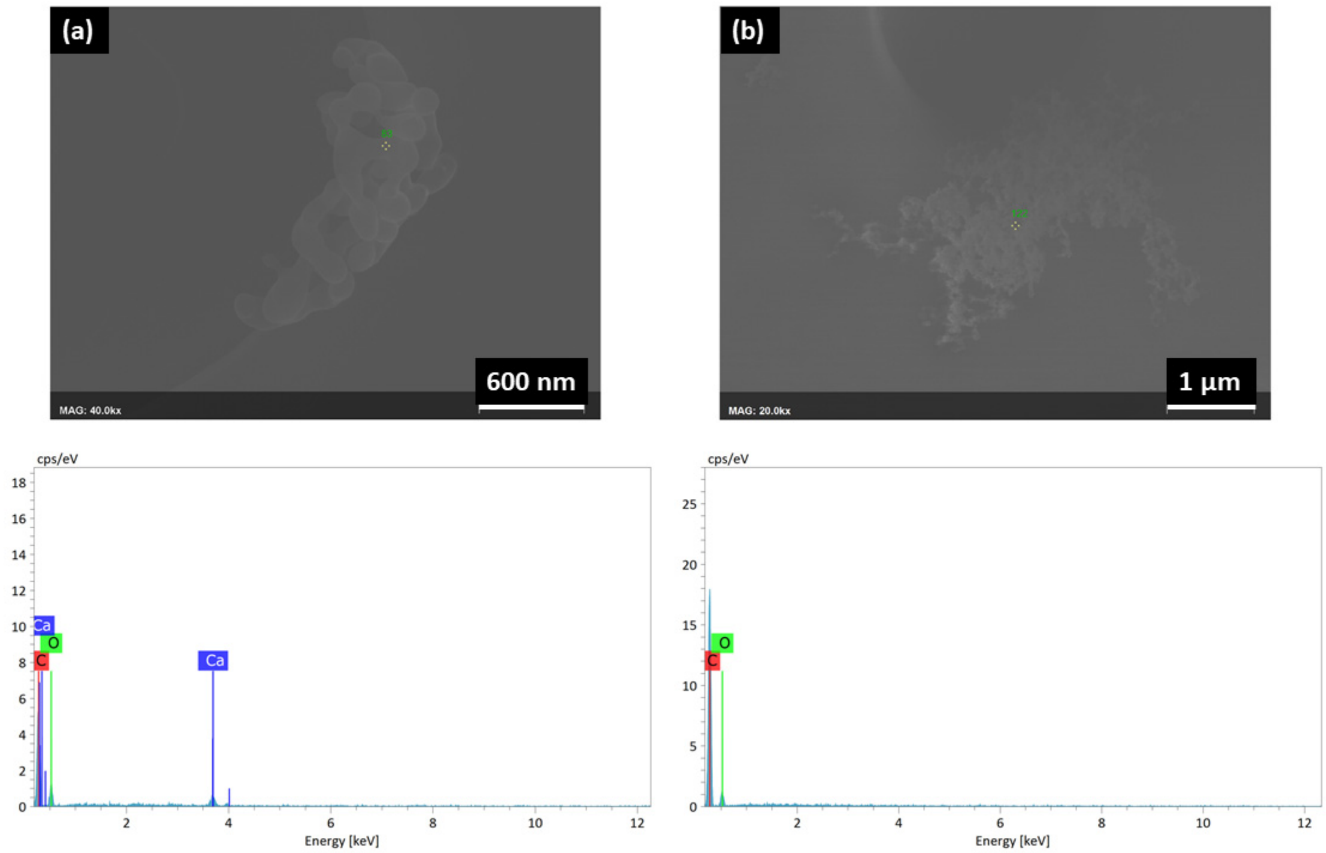

Figure S6. Scanning electron microscopy images and energy dispersive X-ray analysis spectra of particles released during the resin recovery task: (a) nanoscale, agglomerate particles composed of carbon and calcium (SLA process) and (b) nanoscale, branch-chain particles composed of carbon (DLP process).

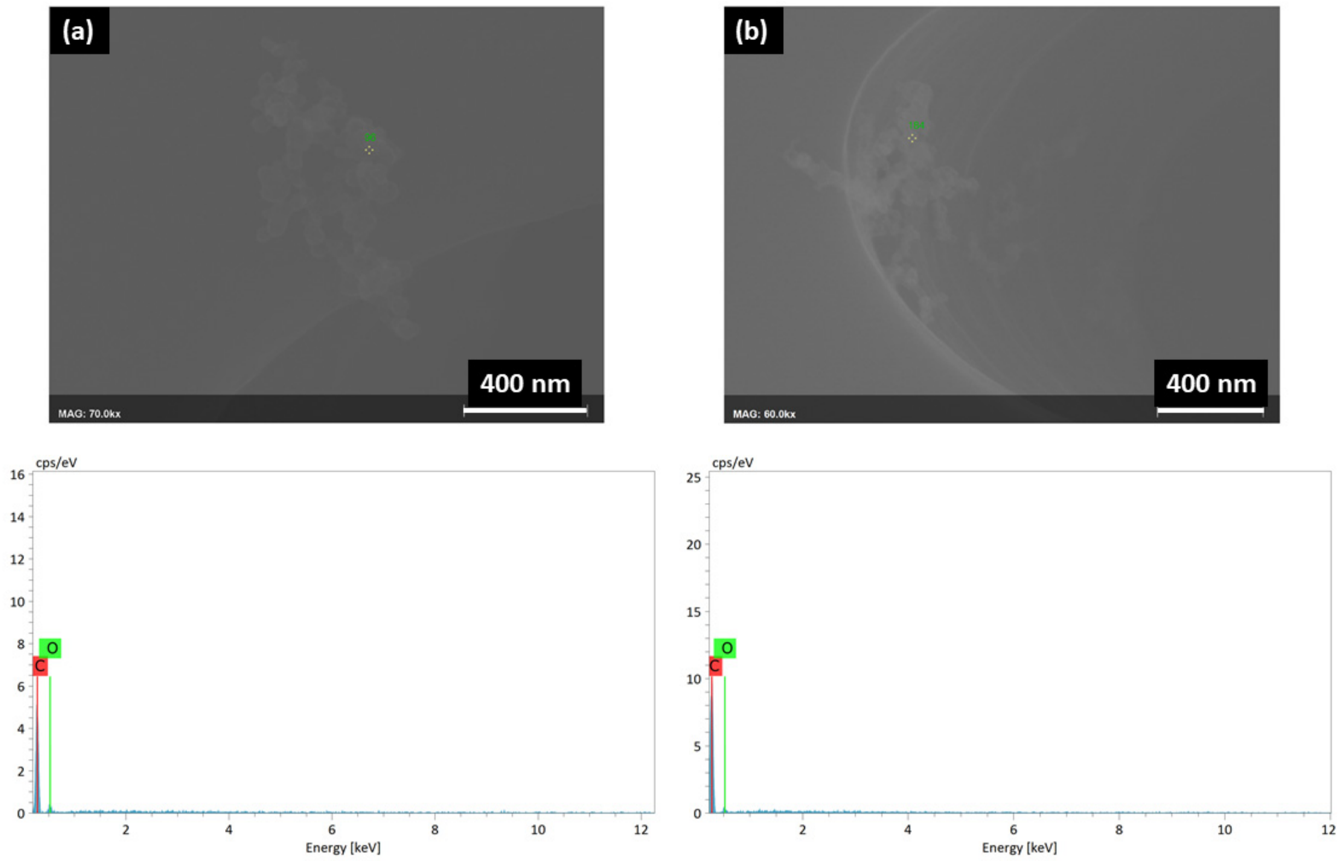

Figure S7. Scanning electron microscopy images and energy dispersive X-ray analysis spectra of particles released during the printed object curing task: (a) and (b) nanoscale, branch-chain particles released from the SLA and DLP processes, respectively. These particles were composed of carbon.

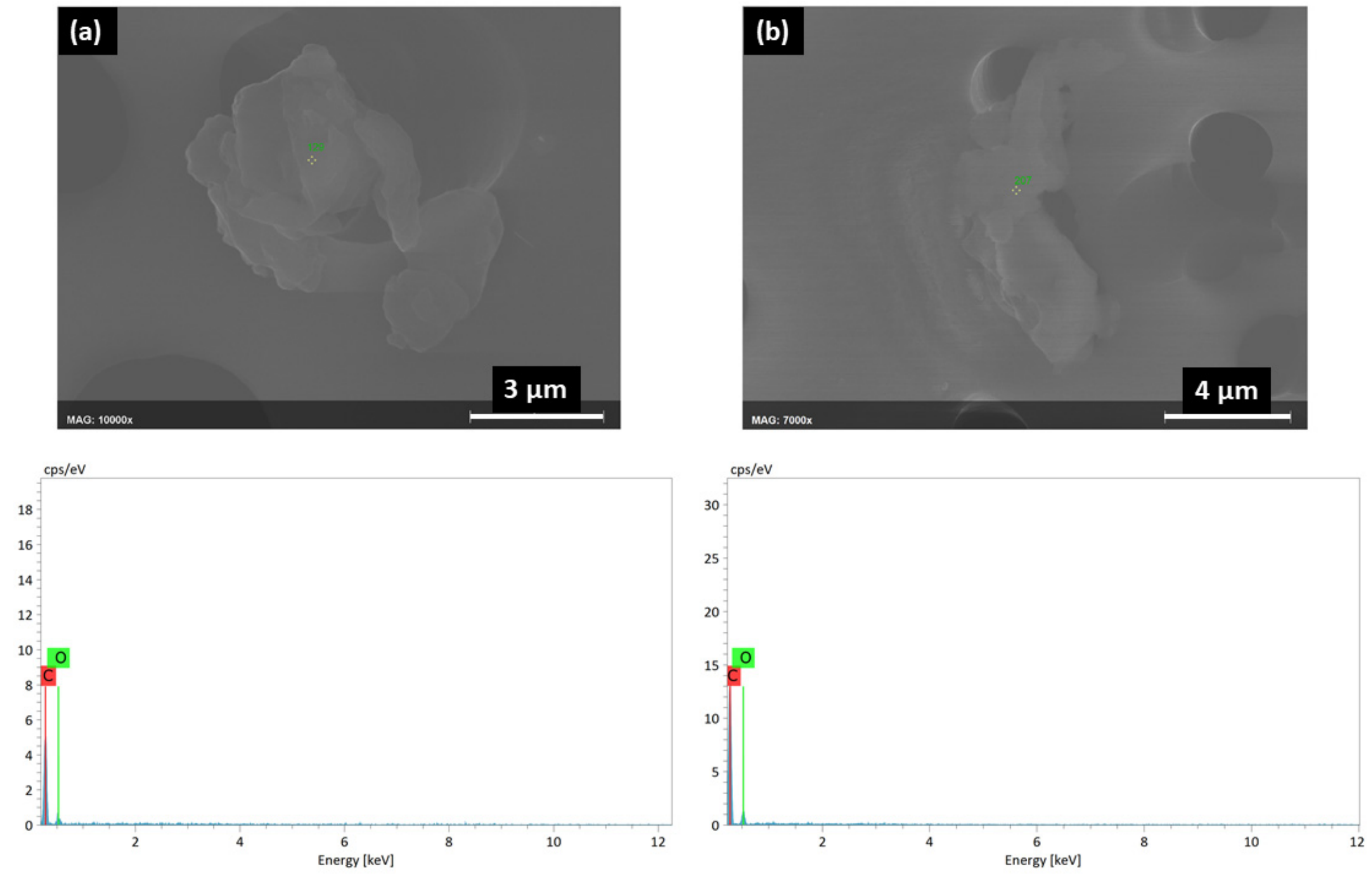

Figure S8. Scanning electron microscopy images and energy dispersive X-ray analysis spectra of particles generated during the sanding task: (a) and (b) micronscale, irregular-shaped particles released from the SLA and DLP processes, respectively.
